# Reassortant H5N1 Avian Influenza Virus Bearing PB2 Gene From a 2009 Pandemic H1N1 Exhibits Increased Pathogenicity in Mice

**DOI:** 10.3389/fmicb.2018.00631

**Published:** 2018-04-03

**Authors:** Xian Lin, Shiman Yu, Kelei Guo, Xin Sun, Haiming Yi, Meilin Jin

**Affiliations:** ^1^State Key Laboratory of Agricultural Microbiology, College of Veterinary Medicine, Huazhong Agricultural University, Wuhan, China; ^2^Key Laboratory of Preventive Veterinary Medicine in Hubei Province, The Cooperative Innovation Center for Sustainable Pig Production, Wuhan, China; ^3^Key Laboratory of Development of Veterinary Diagnostic Products, Ministry of Agriculture of the People’s Republic of China, Wuhan, China; ^4^International Research Center for Animal Disease, Ministry of Science and Technology of the People’s Republic of China, Wuhan, China

**Keywords:** influenza virus, reassortment, H5N1, A(H1N1)pdm2009, PB2, pathogenicity

## Abstract

Reassortment is a key driving force of the evolution and host adaptation of the influenza virus. A(H1N1)pdm2009 (pdm09), a novel H1N1 influenza viral subtype, caused a pandemic in 2009. The strain was established in pig herds and cocirculated with the highly pathogenic H5N1 avian influenza virus. The coexistence of pdm09 with H5N1 raises concerns that reassortment may cause the development of novel viral strains with unpredictable virulence. Given that the viral polymerase subunit PB2 is a determinant of host range and pathogenicity, and that the substantial amino acid differences in PB2 between pdm09 and H5N1, including positions 590/591 and 271, which are shown to play key roles in enhanced polymerase activity in mammalian host cells, we generated a reassortant virus containing PB2 derived from a pdm09 (A/Liaoning/1/2009, LN/09) to investigate if pdm09-derived PB2 can function in a heterologous avian virus isolate as an adaptive strategy, with H5N1 (A/duck/Hubei/hangmei01/2006, HM/06) as the backbone. We assessed the biological characteristics, including pathogenicity, replication, and polymerase activity, of the reassortant. Compared with HM/06 and LN/09, H5N1 hybrid virus containing PB2 from LN/09 exhibited significantly increased pathogenicity in mice and proliferation activity in mammalian cell lines, as well as markedly enhanced polymerase activity. Our results indicate that the coexistence of H5N1 and pdm09 may pose a great threat to public health through reassortment. Moreover, our results highlight the importance of monitoring the emergence of H5N1 reassortants containing pdm09-derived PB2.

## Introduction

Reassortment is an important mechanism for the generation of novel influenza strains capable of causing pandemics. For example, influenza subtypes that caused pandemics in 1957, 1968, and 2009 emerged through genetic recombination (Hsieh et al., 2006; Smith et al., 2009). H7N9, which emerged in China in 2013, is also derived from reassortment events (Liu et al., 2013).

Since 1997, highly pathogenic avian influenza (HPAI) viruses of the H5N1 subtype have spread around the globe and have become global public health concern. The dissemination of these viruses is accompanied by the occasional transmission of HPAIV H5N1 viruses to humans (Neumann et al., 2010). H5N1 frequently jumps into pig populations (Nidom et al., 2010; He et al., 2013). Some human H3N2 and H1N1 subtypes also can infect pigs (Hiromoto et al., 2012). In April 2009, a novel swine-origin H1N1 subtype (pdm09) caused a pandemic. pdm09 is highly transmissible and can establish infection in a wide range of hosts (Peiris et al., 2009). A growing body of evidence has shown that pdm09 has been established in humans and pigs and undergone further reassortment with other influenza subtypes (Chen et al., 2013; Grontvedt et al., 2013; Lange et al., 2013). The coexistence of HPAIV H5N1 with pdm09 provides the potential for reassortment, which poses a massive threat to public health through the generation of highly pathogenic viral strains. Therefore, the potential risk posed by reassortment necessitates the improved understanding of the specific genetic factors that contribute to the pathogenicity of influenza viruses.

PB2, a viral polymerase subunit, is a well-known determinant of host range and pathogenicity (Hatta et al., 2001). The PB2 of human-origin influenza strains predominantly possesses a lysine at the 627 position (627K), whereas that of avian-origin influenza strains often possesses glutamic acid at the 627 position (627E) (Subbarao et al., 1998; Hatta et al., 2001). PB2 627K has higher activity than PB2 627E, and the increased activity of viral polymerases is associated with increased viral replication and pathogenicity (Subbarao et al., 1993; Rameix-Welti et al., 2009). Although pdm09 expresses the PB2 gene with 627E, a 590/591 SR polymorphism facilitates its evasion of host restriction in human cells (Mehle and Doudna, 2009). Besides, pdm09 express 271A in PB2, which plays a key role in enhanced polymerase activity in mammalian host cells. By contrast, HPAIV H5N1 express 627E and 271T in PB2, and do not possess the SR polymorphism in 590/591. Duo to the substantial amino acid differences in PB2 between pdm09 and H5N1, and the potential reassortant risk of coexistence of pdm09 with H5N1, it is attractive to investigate if pdm09-derived PB2 can function in a heterologous HPAIV H5N1 virus isolate. Although several studies have previously investigated the compatibility between avian H5N1 and other human influenza viruses, including H3N2 (Chen et al., 2008; Li et al., 2010) and pdm09 (Octaviani et al., 2010), no study has focused on the functions of the PB2 gene from pdm09 in HPAIV H5N1. Thus, investigating the contribution of pdm09-derived PB2 to the pathogenicity and replication capability of an H5N1 hybrid strain is of great importance.

Here, we created H5N1 reassortant virus containing pdm09-derived PB2. We then evaluated the virulence of the reassorted virus. Our *in vitro* and *in vivo* studies indicated that pdm09-derived PB2 can enhance the pathogenicity of H5N1 reassortant virus in mice. Furthermore, our results highlighted the importance of monitoring the emergence of H5N1 reassortant virus with pdm09-derived PB2.

## Materials and Methods

### Ethics Statement

This study was conducted in strict accordance with the recommendations provided in the Guide for the Care and Use of Laboratory Animals of the Ministry of Science and Technology of the People’s Republic of China. Animal experiments were approved by the Hubei Administrative Committee for Laboratory Animals (Approval No. SYXK-2010-0029). All experiments with live viruses and animals were performed in a biosafety level 3 animal facility and complied with the instructions of the institutional biosafety manual.

### Viruses and Cells

A/duck/Hubei/hangmei01/2006(H5N1) (HM/06) and A/Liaoning/1/2009(H1N1) (LN/09) were conserved in our laboratory, and the NCBI accession numbers for the viruses HM/06 and LN/09 are EU594346–EU594353 and JX403975–JX403982 respectively. Viral titers were determined through 50% tissue culture infectious dose (TCID_50_) analysis in Madin–Darby canine kidney cells (MDCKs). MDCK, the human lung carcinoma cell line A549, and 293T cells were obtained from CTCC (Wuhan, China). Cells were maintained in Dulbecco’s modified Eagle’s medium supplemented with 10% heat-inactivated fetal bovine serum (FBS) (Hyclone, Logan, UT, United States) and incubated in a humidified incubator at 37°C with 5% CO_2_.

### Reverse Genetics

H5N1 hybrid virus expressing PB2 from LN/09 (here and after named LN_PB2_), in which only PB2 gene of H5N1 was replaced with PB2 gene from LN/09, was generated using the eight-plasmid system as described previously (Hoffmann et al., 2000) and confirmed through sequence analysis. Briefly, a mixture of 293T and MDCK cells were co-transfected with PHW2000 plasmids encoding all eight influenza virus segments using Lip2000 (Invitrogen). After 48 h, the supernatants were harvested and inoculated in to 10-day-old SPF embryonated chicken eggs for virus propagation. Then, viral RNA was extracted and analyzed by RT-PCR. Each viral gene was confirmed through sequence analysis.

### Viral Replication Test

Madin–Darby canine kidney cell and A549 cells were infected at a multiplicity of infection (MOI) of 0.1 for 1 h at 37°C. Cells were washed thrice to remove unbound viruses and cultured in DMEM containing 0.1% FBS with 0.5 μg/ml L-1-tosylamido-2-phenylethyl chloromethyl ketone (TPCK)-treated trypsin. HM/06 and LN_PB2_ were cultured in the absence of TPCK trypsin. Cell supernatant samples were collected at 12, 24, 36, 48, and 60 h post-infection (hpi) for viral titer determination through TCID_50_ assay in MDCK cells.

### Animal Experiment

The 50% mouse lethal dose (MLD_50_) values were determined by intranasally inoculating groups of five 6 to 8-week-old female BALB/c mice with 50 μl of 10-fold serial dilutions of the viruses. To determine morbidity and mortality rates, groups of 6 to 8-week-old female BALB/c mice were intranasally challenged with PBS Control or 100 TCID_50_ units of HM/06, LN/09, or the reassortant LN_PB2_ in 50 μl volumes. Mouse body weight and survival were recorded daily for 14 days. At the indicated days post-infection (dpi), three control and three infected mice were euthanized. Lungs, brains, hearts, livers, spleens, and kidneys were collected from the euthanized mice and immediately stored at –80°C until used for viral titer and cytokine detection. Samples were homogenized in 1 ml of cold PBS. Viral titers were determined from clarified sample homogenates through TICD_50_ analysis. Meanwhile, cytokine (IL-1α, IL-6, MCP-1, and KC) levels were detected in lung homogenates using the Bioplex Protein Assay system (Bio-Rad, Hercules, CA, United States) in accordance with the manufacturer’s instructions. To measure macrophage and neutrophil content in lungs, whole lung cells were prepared in DMEM with 2% fetal calf serum following digestion with collagenase–DNase (Sigma-Aldrich, St. Louis, MO, United States) at 37°C and manual disruption (Baumgarth et al., 1994). Red blood cells were removed through lysis buffer (Beyotime, Shanghai, China) treatment. Lung cells were resuspended in PBS and then stained overnight at 4°C to reduce non-specific reaction with fluorophore-conjugated antibodies (BD Bioscience) specific for macrophages (CD11b-FITC) and neutrophils (Ly6G/C-PE). Cells were then washed twice and fixed with 4% paraformaldehyde at room temperature for 30 min. Flow cytometry was performed with BD FACSCalibur system, and BD CellQuest was used to analyze macrophage and neutrophil contents.

### Polymerase Activity Analysis

A dual-luciferase reporter assay system (Promega) was used to measure polymerase activities. To generate each of the 16 possible RNP complex combinations, 0.5 μg of the luciferase report plasmid pPoll-NP-Luc (pNP-Luc), which was kindly provided by Dr. Hualan Chen (Harbin Veterinary Research Institute), and 20 ng of the internal control plasmid *Renilla* (pGL4.75 hRluc/CMV) (Promega) were co-transfected into 293T cells together with 0.5 μg each of plasmid PHW2000 expressing PB2, PB1, PA, or NP derived from HM/06 or LN/09. Cells were lysed 36 h after transfection, and firefly and *Renilla* luciferase activities were measured in accordance with the manufacturer’s instructions. Values were reported as the results of three independent experiments and were standardized to the activities of HM/06-derived polymerase.

### Statistical Analysis

Viral loads and polymerase activity values were expressed as mean ± SD. The statistical significance of differences between experimental groups was determined through two-way ANOVA, Student’s *t*-test, or Log-rank test. Differences were considered significant at *P* < 0.05.

## Results

### H5N1 Hybrid Virus Containing pdm09-Derived PB2 Replicate Efficiently in Mammalian Cells

**FIGURE 1:**
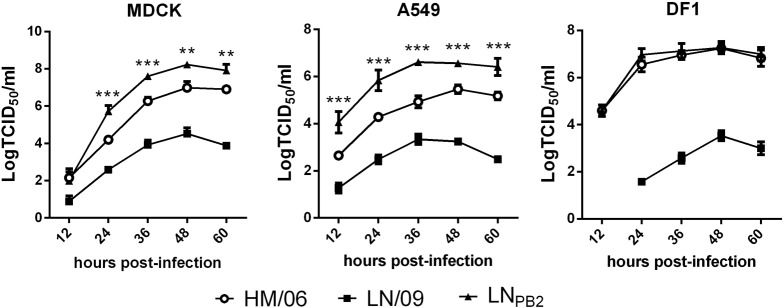
Growth curves of reassortant H5N1 and parental viruses. Viral replication abilities in MDCK, A549, and DF1 cells were determined on the basis of TCID_50_ at indicated time points. Data are presented as the means ± SD of three independent experiments. Statistical significance of differences is analyzed by two-way ANOVA. ^∗∗^*P* < 0.01, ^∗∗∗^*P* < 0.001.

PB2 is involved in host range restriction and pathogenicity. Given that pdm09 cocirculates with avian H5N1 viruses in many areas of the world, we used reverse genetics to generate reassortant virus LN_PB2_. LN_PB2_ contains PB2 gene from pdm09 virus strain LN/09, and other genes (PB1, PA, HA, NP, NA, M, and NS) from HM/06, a HPAIV H5N1 subtype. Due to the substantial amino acid differences between PB2 of HM/06 and LN/09 (**Table 1**), we then assessed the replication capacity of the reassortant LN_PB2_ in three different cell lines (MDCK, A549, and DF1) compared with the parent viruses. MDCK cells are the standard for determining the replication capacity of influenza viruses. A549 cells are the primary target cell types of the influenza virus, and DF1 has an avian origin. Cells were infected at an MOI of 0.1 with the two parental or reassortant virus, and viral titers were determined at indicated hpi through TCID_50_ analysis. In all the studied cell lines, HM/06 exhibited efficient replication, whereas LN/09 exhibited weak replication (**Figure 1**). Notably, compared with the replication capacity of both of its parental viruses, the replication capacity of the reassortant LN_PB2_ remarkably increased in MDCK and A549 cells but not in DF1 cells. The results indicated that LN/09 PB2 can promote the replication of HM/06 in mammalian cells.

**Table 1:** Amino acid differences between PB2 of HM/06 and LN/09 viruses.

Virus	Residue in PB2																
	**54**	**64**	**65**	**76**	**89**	**108**	**126**	**147**	**184**	**225**	**271**	**292**	**295**	**299**	**309**	**315**	**339**

HM/06	K	T	E	A	L	A	K	I	I	S	T	I	V	K	G	M	T
LN/09	R	M	D	T	V	T	N	T	A	G	A	V	M	R	D	I	K

**Virus**	**Residue in PB2**																

	**340**	**369**	**387**	**453**	**467**	**477**	**468**	**483**	**495**	**559**	**576**	**588**	**590**	**591**	**645**	**649**	**684**

HM/06	K	K	S	P	L	R	V	V	I	T	G	A	G	Q	M	I	T
LN/09	N	R	N	S	M	G	I	M	V	I	E	T	S	R	L	V	S

### Reassortant LN_PB2_ Exhibits Increased Pathogenicity in Mice

**FIGURE 2:**
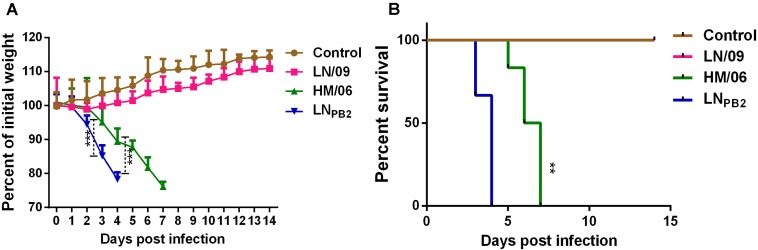
Pathogenicity of reassortant H5N1 and parental viruses in mice. At 6–8 weeks of age, groups of female mice were infected with 100 TCID_50_ of the indicated viruses or PBS control. **(A)** Nine mice each groups were monitored daily for body weight, statistical significance of differences is analyzed by two-way ANOVA. **(B)** Six mice each groups were monitored daily for survival, statistical significance of difference is analyzed by Log-rank test. Data are presented as means ± SD. ^∗∗^*P* < 0.01, ^∗∗∗^*P* < 0.001.

To study the effects of pdm09-derived PB2 on the virulence of H5N1 *in vivo*, we first determined the MLD_50_ of the parental and reassortant viruses. We found that substituting the PB2 gene in HM/06 with LN/09-derived PB2 sharply increases the pathogenicity of the reassortant virus in mice. LN_PB2_ had an MLD_50_ of 10^1.5^ TCID_50_, whereas HM/06 had an MLD_50_ of 10^3.5^ TCID_50_ and LN/09 caused non-lethal infection. To further investigate the virulence of the reassortant virus, we recorded the weight and survival rates of mice infected with the parental or reassortant viruses. As shown in **Figure 2A**, HM/06-infected mice exhibited significant weight loss at 3 dpi. LN/09-infected mice exhibited negligible weight loss during 1 and 2 dpi. LN_PB2_ challenge resulted in more rapid weight loss than HM/06 and LN/09 challenge. Specifically, LN_PB2_ challenge caused a drastic weight loss of more than 20% at 4 dpi. Although HM/06- and LN_PB2_-infected mice all died over the course of infection, LN_PB2_-infected mice began to die at 3 dpi. The time-to-death of LN_PB2_-infected mice is 2 days faster than that of HM/06-infected mice. All LN_*PB2*_-infected mice succumbed by 4 dpi, 3 days earlier than HM/06-infected mice. As expected, LN/09 did not cause lethal infection (**Figure 2B**). These results clearly demonstrated that PB2 derived from LN/09 (pdm09) significantly increases the virulence of the H5N1 recombinant virus in mice.

### pdm09-Derived PB2 Increases Viral Titers in Tissues from Mice Infected with Reassortant LNPB2

**FIGURE 3:**
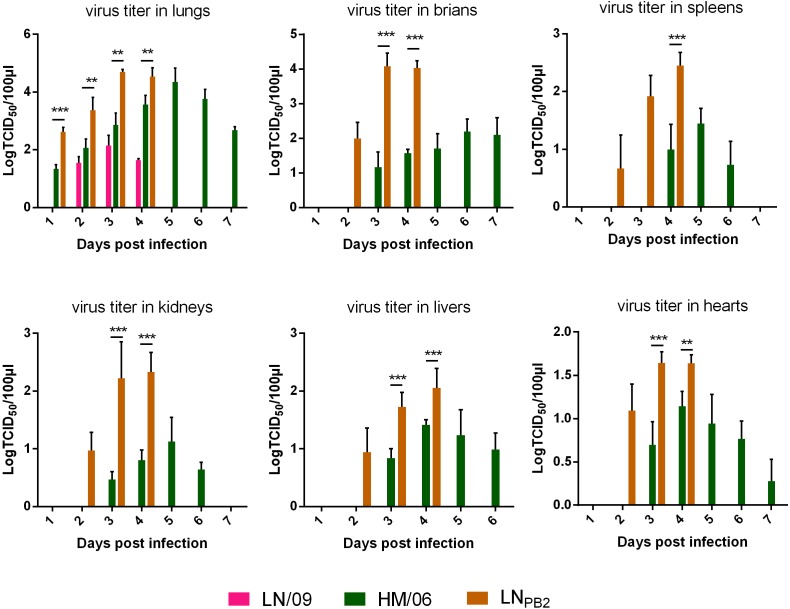
Viral titers in tissues from infected mice. Twenty four mice each groups were infected with 100 TCID_50_ of the indicated viruses. Three mice were euthanized per group for tissue collection in indicated dpi (Lungs of LN_PB2_-infected mice were not collected at 5 dpi, because all LN_PB2_-infected mice died at 4 dpi). Viral titers were determined on the basis of TCID_50_. Data are presented as means ± SD. Statistical significance of differences is analyzed by two-way ANOVA. ^∗∗^*P* < 0.01, ^∗∗∗^*P* < 0.001.

To investigate the potential mechanism underlying the drastically increased pathogenicity of LN_PB2_ in mice, we systemically explored the proliferation of the parental virus and the reassortant LN_PB2_ in mouse organs, including lungs, brains, hearts, kidneys, spleens, and livers, over the period of 1–7 dpi (**Figure 3**). LN/09 could only be detected in lungs at 2–4 dpi. Furthermore, viral titers peaked at 10^2.15^ TCID_50_/100 μl at 3 dpi and declined at 4 dpi. HM/06 viruses were recovered from all organs, and exhibited the highest viral replication ability in the lungs. HM/06 viral titers in the lungs continuously increased from 2 dpi, peaked at 10^4.35^ TCID_50_/100 μl at 5 dpi, and declined at 6 dpi. By contrast, at 4 dpi, LN_PB2_ titers in lungs were significantly higher than HM/06 titers (LN_PB2_-infected mice all died within 4 days) and reached a maximum value of 10^4.69^ TCID_50_/100 μl at 3 dpi, 2 days earlier than HM/06. Additionally, at 4 dpi, viral titers in the spleens, kidneys, livers, and hearts of LN_PB2_-infected mice were all higher than in those of HM/06-infected mice. Given that HM/06 is a HPAIV exhibiting neurotropism and significant neurovirulence (Zou et al., 2009), analyzing the viral titers of the brains of HM/06-infected mice is of great interest. As shown in **Figure 3**, H1N1 was not detected in brains. HM/06 were detected in brains at titers that slowly but gradually increased from 3 to 7 dpi. Surprisingly, LN_PB2_ was recovered from brains by as early as 2 dpi at a titer of 10^2.00^ TCID_50_/100 μl. At 3 dpi, the LN_PB2_ titer sharply increased to 10^4.08^ TICD_50_/100 μl, which is almost 8400-fold higher than the HM/06 titer at the same time point. We could not detect viral titers on 5 and 7 dpi because all LN_PB2_-infected mice died on 4 dpi. These results indicated that LN/09-derived PB2 substantially promotes the replication capacity of the reassortant H5N1. This effect may contribute greatly to the increased virulence of H5N1 in mice.

### LN_PB2_ Elicits High Levels of Proinflammatory Cytokines in the Lung

**FIGURE 4:**
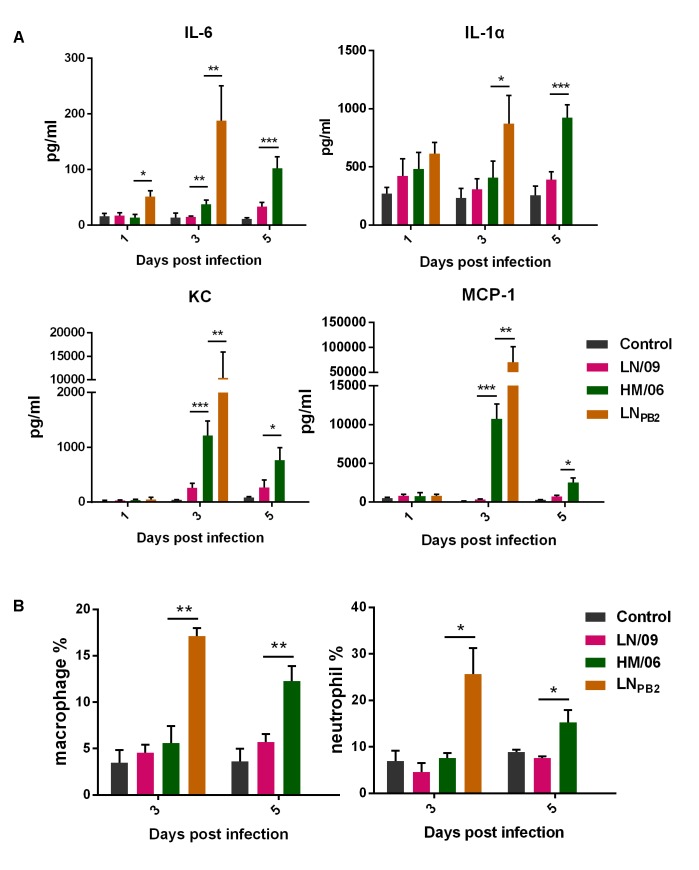
Proinflammatory response in lungs. Lungs were collected from infected mice at indicated dpi (Lungs of LN_PB2_-infected mice were not collected at 5 dpi, because all LN_PB2_-infected mice died at 4 dpi). The contents of IL-6, IL-1α, KC, and MCP-1 **(A)** and numbers of macrophages and neutrophils **(B)** were detected as described in Section “Materials and Methods.” Data are presented as means ± SD. Statistical significance of difference is analyzed by two-way ANOVA. ^∗^*P* < 0.05, ^∗∗^*P* < 0.01, and ^∗∗∗^*P* < 0.001.

Many studies have implicated host innate immune responses in the high pathogenicity of H5N1. We thus analyzed the contents of several key cytokines and chemokines (IL-6, IL-1α, KC, and MCP-1) in the lungs of mice at 1, 3, and 5 dpi (except LN_PB2_-infected group). As shown in **Figure 4A**, at 1 dpi, lungs from LN_PB2_-infected mice exhibited higher titers of IL-6 than lungs from HM/06- and LN/09-infected mice. By contrast, lungs from LN_*PB2*_, HM/06, and LN/09-infected mice exhibited similar IL-1α, KC, and MCP-1 titers. However, the IL-6, KC, and MCP-1 titers of lungs from all infected mice increased on 3 dpi compared with those on 1 dpi. Interestingly, IL-6, IL-1α, KC, and MCP-1 titers are all significantly higher in the lungs of LN_PB2_-infected mice than in those of HM/06-infected mice. Unfortunately, we could not measure the cytokine and chemokine contents of LN_PB2_-infected mice at 5 dpi because all the mice died at 4 dpi. The results suggested that that the proinflammatory response induced by LN_PB2_ infection is more intense than that induced by HM/06 and LN/09 infection.

Severe H5N1 infection can lead to the rapid infiltration of high numbers of macrophages and neutrophils into the lungs; this response has an important role in acute lung inflammation, which is associated with pathogenicity (Perrone et al., 2008). Thus, we investigated the macrophage and neutrophil content of the lungs of infected mice (**Figure 4B**). On 3 dpi, the macrophage and neutrophil contents of LN_PB2_-infected lungs were significantly higher than those of HM/06- and LN/09-infected lungs; this result is consistent with the tested cytokine and chemokine content (**Figure 4A**). On 5 dpi, HM/06 infection induced the production of higher numbers of macrophages and neutrophils than LN/09 infection. These data demonstrated that infection with LN_PB2_ can induce more intense macrophage and neutrophil infiltration to the lungs than infection with other viruses. This response could enhance proinflammatory responses and increase viral pathogenicity in mice.

### LN/09 PB2 Enhances the Polymerase Activity of H5N1 Recombinants

**FIGURE 5:**
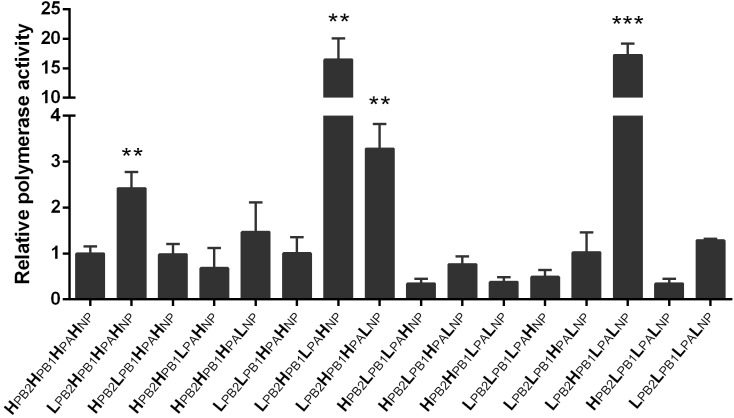
Polymerase activities of 16 RNP complexes. 293T cells were co-transfected with PHW2000 expressing LN/09- or HM/06-derived PB2, PB1, PA, or NP and with the luciferase report plasmid pNP-Luc and the internal control plasmid *Renilla* (pGL4.75 hRluc/CMV). After 36 h post-transfection, cells were lysed to measure pNP-Luc and *Renilla* luciferase activities. Values shown represent the means ± SD of the results of three independent experiments and are standardized to HM/06 values. The statistical significance of differences is analyzed with Student’s *t*-test. ^∗^, Compared with the value of HM/06 (H_PB2_H_*PA*_H_*PB1*_H_*NP*_); ^∗∗^*P* < 0.01, ^∗∗∗^*P* < 0.001.

Polymerase activity has important associations with viral replication, pathogenicity, and host adaptation (Neumann and Kawaoka, 2006). We performed a luciferase assay to investigate the polymerase activity of 16 different ribonucleoprotein (RNP) complex combinations from LN/09 and HM/06 at 37°C in 293T cells (**Figure 5**). The data indicated that the activity of the pandemic H1N1 LN/09 polymerase (L_PB2_L_*PB1*_L_*PA*_L_*NP*_; “L” stands for the LN/09 virus) is slightly higher than that of the avian H5N1 HM/06 polymerase (H_PB2_H_*PB1*_H_*PA*_H_*NP*_; “H” stands for the HM/06 virus). Interestingly, the polymerase activity of the RNP combination consisting of LN/09-derived PB2 and HM/09-derived PB1, PA, and NP (L_PB2_H_*PB1*_H_*PA*_H_*NP*_) significantly increased compared with that of LN/09 and HM/09. Remarkably, the reassortant LN_PB2_ possesses this type of RNP combination. Additionally, two RNP combinations (L_PB2_H_*PB1*_L_*PA*_H_*NP*_ and L_PB2_H_*PB1*_L_*PA*_L_*NP*_), which exhibited the highest polymerase activities among all combinations, possess LN/09-derived PB2 and PA. The removal of PB2 drastically decreased polymerase (H_PB2_H_*PB1*_L_*PA*_H_*NP*_ and H_PB2_H_*PB1*_L_*PA*_L_*NP*_) activities to levels equivalent to or lower than those of HM/06. These results indicate that LN/09-derived PB2 may play a critical role in RNP complexes compatibility between HM/06 and LN/09.

## Discussion

Reassortment is an important mechanism for the evolution of influenza viruses. This phenomenon could lead to antigenic shift and generate pandemic strains. The coexistence of pdm09 and H5N1 viruses provides an opportunity for the emergence of novel viral subtypes with considerable potential threats to public health. Several studies have previously investigated the compatibility between avian H5N1 and other human influenza viruses, including H3N2 (Chen et al., 2008; Li et al., 2010) and pdm09 (Octaviani et al., 2010). However, no study has focused on the roles of the PB2 gene in reassortment. PB2 is an important determinant of host range restriction and virulence in animal models (Hatta et al., 2001). Glutamic acid is present at position 627 (627E) in the avian PB2, whereas lysine is present at position 627 (627K) in human PB2. PB2 627K is associated with enhanced polymerase activity and viral replication, transmission, and pathogenicity in mammals. Interestingly, although pdm09 possesses the avian-signature 627E, this virus is efficiently transmitted in humans and animal models (Fraser et al., 2009) because it has acquired second-site suppressor mutations in its PB2 that confer enhanced polymerase activity (Mehle and Doudna, 2009).

In this study, we demonstrated that replacing HM/06 (H5N1)-derived PB2, which encodes 627E, by LN/09 (pdm09) promotes viral replication capacity in mammalian cells (**Figure 1**) and virulence in mice (**Figures 2–4**), which may be attributed to the following mechanisms: (1) LN/09-derived PB2 can significantly increase the polymerase activity of the reassortant virus (**Figure 5**); (2) LN/09-derived PB2 expands the tissue tropism of the H5N1 recombinant (**Figure 3**); (3) LN/09-derived PB2 is highly compatible with other genes of HM/06, which may contribute great to genome transcript, replication, and virus assembly. Additionally, other amino acids difference except 590/591 may also play some roles in the pathogenicity. It is interesting to investigate that if PB2 of pdm09 can also increase the pathogenicity of H5N1 recombinant when the amino acids 590/591(S/R) were mutated to 590/591(G/Q) in the future, which are same as those in H5N1. Previous studies suggested that H9N2 PB2 significantly decreases the virulence of the H5N1 recombinant in mice (Hao et al., 2016) and significantly decreases the replication capacity of H7N9 (Su et al., 2015). Furthermore, a single PB2 gene substitution between pdm09 PB2 and H9N2 decreases replication and infection ability (Sun et al., 2011). These results indicated that the compatibility of PB2 with remaining genes from other virus may be strain-dependent.

In the present study, the polymerase activity of the RNP complex, which consists of HM/06-derived PB1, PA, and NP and LN/09-derived PB2, significantly increased compared with that of HM/06 and LN/09 (L_PB2_H_*PB1*_H_*PA*_H_*NP*_). However, the polymerase activity of the RNP complex possessing HM/06-derived PB2 and other LN/09-derived genes (H_PB2_L_*PB1*_L_*PA*_L_*NP*_) significantly decreased compared with that of HM/06 and LN/09 (**Figure 5**). This result strongly suggested that LN/09 PB2 is highly compatible with the PB1, PA, and NP of HM/06, whereas HM/06 PB2 is incompatible with the PB1, PA, and NP of LN/09. Furthermore, the reassortant LN_PB2_ virus exhibited a new RNP complex combination (L_PB2_H_*PB1*_H_*PA*_H_*NP*_) with higher activity than HM/06 and LN/09. The L_PB2_H_*PB1*_H_*PA*_H_*NP*_ complex may account for the increased replication capacity in mammalian cells and pathogenicity in mice of the reassortant LN_PB2_ virus. It is confusing that Octaviani found that pdm09 PB2 could low the polymerase activity of a H5N1 strain in a previous study (Octaviani et al., 2010), which is in contrast to our result. Our analysis suggests there are four amino acids difference between A/California/04/2009 (H1N1) used in Octaviani’s study and LN/09 in the present study. Although we are fail to get the sequence information of A/Vietnam/HN31604/2009 (H5N1) virus used in Octaviani’s study, we speculate that there are likely many differences between the H5N1 strains used in the two studies, due to the different time, region, and species for virus isolation. The substantial amino acid differences between the viruses used may make the results different, which needs further study. Surprisingly, two different RNP complexes (L_PB2_H_*PB1*_L_*PA*_H_*NP*_ and L_PB2_H_*PB1*_L_*PA*_L_*NP*_) with LN/09-derived PB2 have nearly 17-fold higher activity than HM/06. Even interestingly, these two RNP complexes contain a LN/09-derived PA; however, only the PA combination of the RNP complex (H_PB2_H_*PB1*_L_*PA*_H_*NP*_ and H_PB2_H_*PB1*_L_*PA*_L_*NP*_) showed significantly lower activities than the RNP complex of LN_PB2_. This result suggested that PB2 is necessary for high polymerase activity and that the high compatibility between LN/09 PB2 and PA contributes to high polymerase activity. Given these observations, the biological properties of H5N1 hybrids bearing the PB2 and PA of pdm09 require further investigation.

In summary, our data demonstrated that pdm09 PB2 can enhance H5N1 replication in mammalian cells and pathogenicity in mice. Moreover, our data highlighted the importance of monitoring the emergence of H5N1 reassortant virus containing a human-derived PB2.

## Author Contributions

XL and MJ: conceived and designed the experiments and wrote the paper. XL, SY, and XS: performed the experiments. XL, KG, and HY: analyzed the data. KG and HY: contributed reagents/materials.

## Conflict of Interest Statement

The authors declare that the research was conducted in the absence of any commercial or financial relationships that could be construed as a potential conflict of interest.

## References

[B1] Baumgarth N. Brown L. Jackson D. Kelso A. (1994). Novel features of the respiratory tract T-cell response to influenza virus infection: lung T cells increase expression of gamma interferon mRNA in vivo and maintain high levels of mRNA expression for interleukin-5 (IL-5) and IL-10. *J. Virol.* 68 7575–7581. 7933145 10.1128/jvi.68.11.7575-7581.1994 PMC237205

[B2] Chen L. M. Davis C. T. Zhou H. Cox N. J. Donis R. O. (2008). Genetic compatibility and virulence of reassortants derived from contemporary avian H5N1 and human H3N2 influenza A viruses. *PLoS Pathog.* 4:e1000072. 10.1371/journal.ppat.1000072 18497857 PMC2374906

[B3] Chen Y. Zhang J. Qiao C. Yang H. Zhang Y. Xin X. (2013). Co-circulation of pandemic 2009 H1N1, classical swine H1N1 and avian-like swine H1N1 influenza viruses in pigs in China. *Infect. Genet. Evol.* 13 331–338. 10.1016/j.meegid.2012.09.021 23146831

[B4] Fraser C. Donnelly C. A. Cauchemez S. Hanage W. P. Van Kerkhove M. D. Hollingsworth T. D. (2009). Pandemic potential of a strain of influenza A (H1N1): early findings. *Science* 324 1557–1561. 10.1126/science.1176062 19433588 PMC3735127

[B5] Grontvedt C. A. Er C. Gjerset B. Hauge A. G. Brun E. Jorgensen A. (2013). Influenza A(H1N1)pdm09 virus infection in Norwegian swine herds 2009/10: the risk of human to swine transmission. *Prev. Vet. Med.* 110 429–434. 10.1016/j.prevetmed.2013.02.016 23490143 PMC7132443

[B6] Hao X. Hu J. Wang J. Xu J. Cheng H. Xu Y. (2016). Reassortant H5N1 avian influenza viruses containing PA or NP gene from an H9N2 virus significantly increase the pathogenicity in mice. *Vet. Microbiol.* 192 95–101. 10.1016/j.vetmic.2016.07.002 27527770

[B7] Hatta M. Gao P. Halfmann P. Kawaoka Y. (2001). Molecular basis for high virulence of Hong Kong H5N1 influenza A viruses. *Science* 293 1840–1842. 10.1126/science.1062882 11546875

[B8] He L. Zhao G. Zhong L. Liu Q. Duan Z. Gu M. (2013). Isolation and characterization of two H5N1 influenza viruses from swine in Jiangsu Province of China. *Arch. Virol.* 158 2531–2541. 10.1007/s00705-013-1771-y 23836394

[B9] Hiromoto Y. Parchariyanon S. Ketusing N. Netrabukkana P. Hayashi T. Kobayashi T. (2012). Isolation of the pandemic (H1N1) 2009 virus and its reassortant with an H3N2 swine influenza virus from healthy weaning pigs in Thailand in 2011. *Virus Res.* 169 175–181. 10.1016/j.virusres.2012.07.025 22906589

[B10] Hoffmann E. Neumann G. Kawaoka Y. Hobom G. Webster R. G. (2000). A DNA transfection system for generation of influenza A virus from eight plasmids. *Proc. Natl. Acad. Sci. U.S.A.* 97 6108–6113. 10.1073/pnas.100133697 10801978 PMC18566

[B11] Hsieh Y. C. Wu T. Z. Liu D. P. Shao P. L. Chang L. Y. Lu C. Y. (2006). Influenza pandemics: past, present and future. *J. Formos. Med. Assoc.* 105 1–6. 10.1016/S0929-6646(09)60102-9 16440064

[B12] Lange J. Groth M. Schlegel M. Krumbholz A. Wieczorek K. Ulrich R. (2013). Reassortants of the pandemic (H1N1) 2009 virus and establishment of a novel porcine H1N2 influenza virus, lineage in Germany. *Vet. Microbiol.* 167 345–356. 10.1016/j.vetmic.2013.09.024 24139631

[B13] Li C. Hatta M. Nidom C. A. Muramoto Y. Watanabe S. Neumann G. (2010). Reassortment between avian H5N1 and human H3N2 influenza viruses creates hybrid viruses with substantial virulence. *Proc. Natl. Acad. Sci. U.S.A.* 107 4687–4692. 10.1073/pnas.0912807107 20176961 PMC2842136

[B14] Liu D. Shi W. Shi Y. Wang D. Xiao H. Li W. (2013). Origin and diversity of novel avian influenza A H7N9 viruses causing human infection: phylogenetic, structural, and coalescent analyses. *Lancet* 381 1926–1932. 10.1016/S0140-6736(13)60938-1 23643111

[B15] Mehle A. Doudna J. A. (2009). Adaptive strategies of the influenza virus polymerase for replication in humans. *Proc. Natl. Acad. Sci. U.S.A.* 106 21312–21316. 10.1073/pnas.0911915106 19995968 PMC2789757

[B16] Neumann G. Chen H. Gao G. F. Shu Y. Kawaoka Y. (2010). H5N1 influenza viruses: outbreaks and biological properties. *Cell Res.* 20 51–61. 10.1038/cr.2009.124 19884910 PMC2981148

[B17] Neumann G. Kawaoka Y. (2006). Host range restriction and pathogenicity in the context of influenza pandemic. *Emerg. Infect. Dis.* 12 881–886. 10.3201/eid1206.051336 16707041 PMC3373033

[B18] Nidom C. A. Takano R. Yamada S. Sakai-Tagawa Y. Daulay S. Aswadi D. (2010). Influenza A (H5N1) viruses from pigs, Indonesia. *Emerg. Infect. Dis.* 16 1515–1523. 10.3201/eid1610.100508 20875275 PMC3294999

[B19] Octaviani C. P. Ozawa M. Yamada S. Goto H. Kawaoka Y. (2010). High level of genetic compatibility between swine-origin H1N1 and highly pathogenic avian H5N1 influenza viruses. *J. Virol.* 84 10918–10922. 10.1128/JVI.01140-10 20686037 PMC2950597

[B20] Peiris J. S. Poon L. L. Guan Y. (2009). Emergence of a novel swine-origin influenza A virus (S-OIV) H1N1 virus in humans. *J. Clin. Virol.* 45 169–173. 10.1016/j.jcv.2009.06.006 19540800 PMC4894826

[B21] Perrone L. A. Plowden J. K. Garcia-Sastre A. Katz J. M. Tumpey T. M. (2008). H5N1 and 1918 pandemic influenza virus infection results in early and excessive infiltration of macrophages and neutrophils in the lungs of mice. *PLoS Pathog.* 4:e1000115. 10.1371/journal.ppat.1000115 18670648 PMC2483250

[B22] Rameix-Welti M. A. Tomoiu A. Dos Santos Afonso E. van der Werf S. Naffakh N. (2009). Avian Influenza A virus polymerase association with nucleoprotein, but not polymerase assembly, is impaired in human cells during the course of infection. *J. Virol.* 83 1320–1331. 10.1128/JVI.00977-08 19019950 PMC2620912

[B23] Smith G. J. Vijaykrishna D. Bahl J. Lycett S. J. Worobey M. Pybus O. G. (2009). Origins and evolutionary genomics of the 2009 swine-origin H1N1 influenza A epidemic. *Nature* 459 1122–1125. 10.1038/nature08182 19516283

[B24] Su W. Wang C. Luo J. Zhao Y. Wu Y. Chen L. (2015). Testing the effect of internal genes derived from a wild-bird-origin H9N2 influenza a virus on the pathogenicity of an A/H7N9 virus. *Cell Rep.* 12 1831–1841. 10.1016/j.celrep.2015.08.029 26344762

[B25] Subbarao E. K. London W. Murphy B. R. (1993). A single amino acid in the PB2 gene of influenza A virus is a determinant of host range. *J. Virol.* 67 1761–1764. 8445709 10.1128/jvi.67.4.1761-1764.1993 PMC240216

[B26] Subbarao K. Klimov A. Katz J. Regnery H. Lim W. Hall H. (1998). Characterization of an avian influenza A (H5N1) virus isolated from a child with a fatal respiratory illness. *Science* 279 393–396. 10.1126/science.279.5349.393 9430591

[B27] Sun Y. Qin K. Wang J. Pu J. Tang Q. Hu Y. (2011). High genetic compatibility and increased pathogenicity of reassortants derived from avian H9N2 and pandemic H1N1/2009 influenza viruses. *Proc. Natl. Acad. Sci. U.S.A.* 108 4164–4169. 10.1073/pnas.1019109108 21368167 PMC3054021

[B28] Zou W. Yu Z. Zhou H. Tu J. Jin M. (2009). Genetic characterization of an H5N1 avian influenza virus with neurovirulence in ducks. *Virus Genes* 38 263–268. 10.1007/s11262-008-0319-9 19137421

